# Association between the dietary index for gut microbiota and fracture prevalence among women aged 50 years and older: a NHANES-based study with a preliminary exploratory pilot analysis

**DOI:** 10.3389/fpubh.2026.1933908

**Published:** 2026-10-06

**Authors:** Ji Wu, Yunping Liu, Zihao Huang, Xingming Ning, Tao Xu, Qiongxia Yu, Jing Guo, Luming Qi, Zhangmeng Xu

**Affiliations:** 1 Department of Cervicodynia/Omalgia/Lumbago/Sciatica, Sichuan Province Orthopedic Hospital, Chengdu, Sichuan, China; 2 College of Health Preservation and Rehabilitation, Chengdu University of Traditional Chinese Medicine, Chengdu, Sichuan, China; 3 College of Pharmacy, Chengdu University of Traditional Chinese Medicine, Chengdu, Sichuan, China; 4 Department of Foot and Ankle Surgery, Sichuan Province Orthopedic Hospital, Chengdu, Sichuan, China

**Keywords:** bone mineral density, dietary index for gut microbiota (DI-GM), exploratory pilot analysis, fracture prevalence, women aged 50 years and older

## Abstract

**Introduction:**

Gut microbiota has been implicated in bone metabolism, but whether a diet supporting gut microbial health is associated with fracture prevalence in older women remains unclear. This study aimed to examine the association between the dietary index for gut microbiota (DI-GM) and fracture prevalence in women aged 50 years and older.

**Methods:**

We analyzed cross-sectional data from five NHANES cycles, including 6,318 women aged 50 years and older (mean age 63.8 ± 9.6 years). DI-GM scores were derived from 24-h dietary recalls based on 13 dietary components. Self-reported hip, wrist, or spine fracture was the outcome (863 women, 13.7%). Bone mineral density (BMD) was measured by dual-energy X-ray absorptiometry. Weighted logistic regression and exploratory mediation analyses were performed.

**Results:**

Higher DI-GM scores were associated with lower odds of fragility fracture (OR = 0.93, 95% CI: 0.88–1.00, *p* = 0.035) and any fracture (Model 3: OR = 0.96, 95% CI: 0.92–0.99, *p* = 0.045). Neither femoral neck nor spinal BMD significantly mediated these associations. In a small exploratory pilot cohort (*n* = 12), DI-GM scores were positively correlated with spinal BMD (*R* = 0.82, *p* = 0.0011).

**Discussion:**

Higher DI-GM scores were associated with lower fracture prevalence in women aged 50 years and older, although BMD did not significantly mediate this association. Given the cross-sectional design and the small pilot sample, these findings should be considered exploratory and require confirmation in larger prospective studies.

## Graphical abstract

**Figure:**
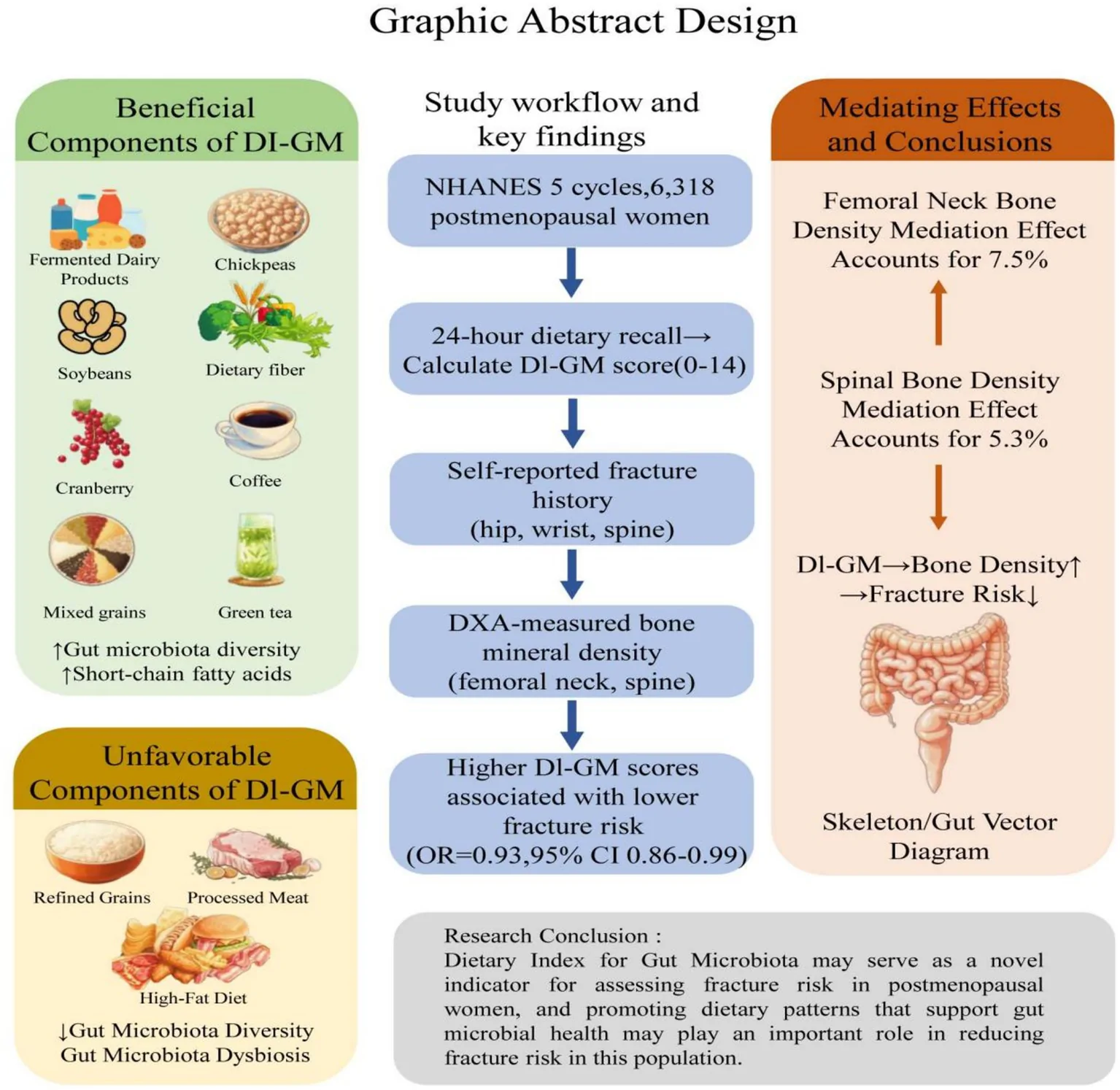
Overall study workflow and key findings.

## 1 Introduction

Fractures, particularly hip, wrist, and vertebral fractures, represent an important public health issue among women aged 50 years and older (1, 2). In women aged 50 years and older, the decline in estrogen levels disrupts bone remodeling homeostasis, resulting in increased bone resorption relative to bone formation, accelerated bone loss, deterioration of bone microarchitecture, and ultimately an elevated prevalence of fracture (3, 4). With the acceleration of population aging, the disease burden of osteoporosis-related fractures continues to increase, leading not only to pain, impaired mobility, disability, and reduced quality of life, but also to substantially increased healthcare costs and mortality (5, 6). Therefore, identifying modifiable, low-cost, and easily implementable factors related to fracture prevalence is of great significance for fracture prevention and public health management in women aged 50 years and older.

Bone mineral density (BMD) is one of the core indicators currently used to assess osteoporosis and fracture prevalence; however, fracture occurrence is not determined by bone mineral density alone. Age, nutritional status, inflammatory state, metabolic abnormalities, muscle function, fall risk, and lifestyle factors may all contribute to fracture susceptibility. In recent years, dietary patterns, as modifiable factors, have attracted increasing attention in the maintenance of bone health and the prevention of fractures (7, 8). Compared with individual nutrients, overall dietary patterns can more comprehensively reflect long-term nutritional exposure and its combined effects on metabolism and inflammation (9, 10).

The dietary index for gut microbiota (DI-GM) is a recently developed, literature-based dietary quality assessment tool designed to evaluate dietary patterns based on food components previously hypothesized to influence gut microbiota health (11). Because gut microbiota is thought to participate in the interplay among diet, metabolism, inflammatory status, and bone health, DI-GM may provide a novel tool for fracture prevalence stratification from a dietary perspective (12, 13). This index incorporates a range of food components considered beneficial or detrimental to gut microbiota, including fermented dairy products, legumes, whole grains, fiber-rich vegetables, fruits, coffee, and green tea, as well as red meat, processed meat, refined grains, and high-fat diets (11). Compared with conventional dietary scores, DI-GM places greater emphasis on the potential regulatory effects of diet on the gut microbial ecosystem, making it particularly suitable for exploring relationships among diet, microbiota-related metabolism, and chronic disease risk. However, previous studies have mainly focused on the associations of individual nutrients, specific food groups, or traditional dietary patterns with BMD and osteoporosis, whereas evidence regarding whether dietary patterns characterized by higher DI-GM scores are associated with fracture prevalence remains limited.

Emerging evidence has begun to apply DI-GM to NHANES populations. A cross-sectional study of 9,805 adults found that higher DI-GM scores were associated with lower DXA-derived visceral adiposity (14). In postmenopausal women aged 50 years and older, higher DI-GM scores were associated with lower odds of osteoporosis, with the highest quartile showing a 44% reduction in odds (15). A mediation analysis further identified caffeine and biological age as mediators of the DI-GM–osteoporosis association, with caffeine mediating 20.33% of the effect (16). However, whether BMD mediates the association between DI-GM and fracture prevalence remains unclear.

Therefore, using data from multiple cycles of the National Health and Nutrition Examination Survey (NHANES), this study included women aged 50 years and older to systematically evaluate the association between DI-GM and the prevalence of hip, wrist, or vertebral fracture, and further explored the potential mediating roles of femoral neck and lumbar spine BMD. In addition, a small exploratory pilot analysis in an independent osteoporosis cohort was conducted to provide preliminary contextual support for the NHANES-based findings. This study aims to provide new cross-sectional epidemiological evidence for fracture prevalence stratification and to inform potential diet-based public health strategies among women aged 50 years and older.

## 2 Materials and methods

### 2.1 Study population

The data for this study were sourced from the National Health and Nutrition Examination Survey (NHANES), a comprehensive survey conducted by the National Center for Health Statistics (NCHS) within the Centers for Disease Control and Prevention (CDC). The survey methodology, including sampling strategies and data collection techniques, is thoroughly described on the CDC’s official website^1^.

For this analysis, we utilized NHANES data from five 2-year cycles: 2005–2006, 2007–2008, 2009–2010, 2013–2014, and 2017–2018. These cycles were selected because they provided detailed measurements of femoral neck and spinal bone density, critical variables for our study objectives. Initially, the combined dataset included 50,463 participants. Participants aged under 50 years (*n* = 36,297), males (*n* = 6,996), and those with missing key variable data (*n* = 852) were excluded from the analysis. Consequently, the final analytical set comprised 6,318 women aged 50 years and older, of whom 863 had a history of fractures (see Figure 1).

**Figure 1:**
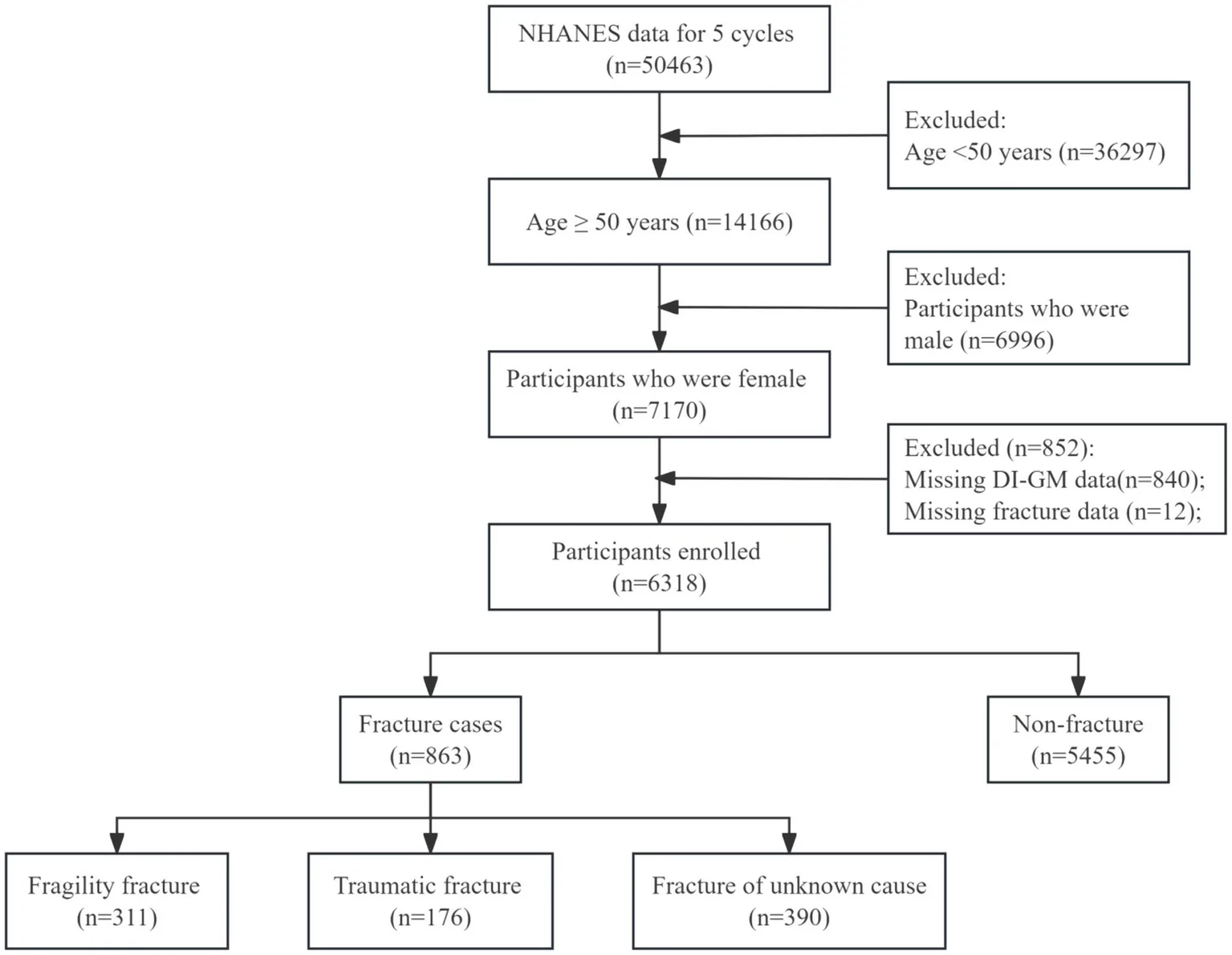
Flowchart of participants inclusion process. Fourteen participants had both fragility fractures and traumatic fractures.

NHANES employs a complex, multistage probability sampling design (17–19). To account for this design and produce nationally representative estimates, we incorporated survey weights, masked variance pseudo-strata (SDMVSTRA), and pseudo-primary sampling units (SDMVPSU) into all primary weighted analyses. Because DI-GM is derived from dietary recall data, we used dietary-specific sample weights following NHANES analytic guidelines. Specifically: (1) WTDR2D was used for participants with two complete and reliable dietary recalls (Day 1 and Day 2); (2) WTDRD1 was used for participants with only one complete and reliable dietary recall (Day 1). Because we combined five 2-year NHANES cycles, we recalibrated the sampling weights by dividing the original 2-year weight by the number of cycles combined (weight/5), in accordance with CDC guidelines for combining multiple NHANES cycles.

### 2.2 Fracture data

Fracture history was collected through self-reported questionnaires, in which participants were asked whether they had ever experienced a fracture in the hip, wrist, or spine. A participant was considered to have a history of fracture if they reported at least one incident in any of these areas (20). To further characterize the fracture history, we classified the first fracture at each site by trauma mechanism using the OSD050 series of variables, which recorded the mechanism of injury for fractures occurring at age 50 years or older. Fractures resulting from “a fall from standing height or less” were defined as fragility fractures. Fractures resulting from a hard fall or a car accident or other severe trauma were classified as traumatic fractures. Fractures with missing or unknown trauma information (coded as 7 or 9) were classified as fractures of unknown cause (21).

### 2.3 DI-GM scores

The DI-GM was calculated based on the method developed by Kase et al. (11). Dietary intake data were obtained from five NHANES cycles (2005–2006, 2007–2008, 2009–2010, 2013–2014, and 2017–2018) using two 24-h dietary recalls. Only participants with reliable dietary recall status were included. For participants with two complete and reliable recalls, we averaged the intakes from both days; for those with only one reliable recall, the single recall was used. The original DI-GM includes 14 components; however, because the FNDDS food codes in NHANES do not distinguish green tea from other tea types, we excluded green tea and used the remaining 13 components—9 beneficial (fermented dairy, chickpeas, soybeans, whole grains, dietary fiber, cranberries, avocados, broccoli, and coffee) and 4 detrimental (red meat, processed meat, refined grains, and high-fat diet). Food classification was performed using FNDDS food codes and food descriptions from the NHANES DRXFCD files, based on food code prefixes and keyword matching. Sex-specific medians for each component were calculated pooled across all five cycles using dietary weights, stratified by sex. For beneficial components, participants with intake > the sex-specific median received 1 point. For detrimental components, participants with intake < the sex-specific median received 1 point. High-fat diet was defined as fat energy percentage ≥ 40% of total calories; participants with < 40% received 1 point. The DI-GM total score ranges from 0 to 13, with higher scores indicating a dietary pattern more favorable to gut microbial health. The DI-GM is a literature-based dietary index hypothesized to influence gut microbial health, rather than a direct measure of gut microbiota composition. The complete DI-GM scoring algorithm is provided in Supplementary File 1.

### 2.4 Bone density measurements

Bone mineral density (BMD, g/cm^2^) was assessed using dual-energy X-ray absorptiometry (DXA) with QDR 4500A fan-beam densitometers (Hologic Inc.) during the Mobile Examination Center visit. Lumbar spine BMD was calculated as the mean BMD of the anteroposterior projection of vertebrae L1–L4. For hip assessments, the left hip was routinely scanned to report total femur, femoral neck, and trochanter BMD; if the participant had undergone left hip arthroplasty or had metal objects in the left hip area, the right hip was scanned instead (22). Exclusion criteria for DXA assessments followed NHANES guidelines.

### 2.5 Covariates

Covariates were selected *a priori* based on clinical expertise and prior literature (23–26). To provide a transparent causal framework, we constructed a directed acyclic graph (DAG) (see Figure 2). The DAG identified the following minimal sufficient adjustment set: diabetes, alcohol consumption, glucocorticoid exposure, hormone therapy, hypertension, physical activity, and smoking. In addition, age, race, and marital status were treated as established confounders and included in all models; they were not explicitly depicted in the DAG to maintain clarity. BMI and vitamin D were not included in the adjustment set because they are not common causes of both DI-GM and fracture; adjusting for them could introduce collider bias. The same covariate adjustment strategy (Models 1–3) was applied consistently across the main analysis, complete-case analysis, and multiple imputation analyses.

**Figure 2:**
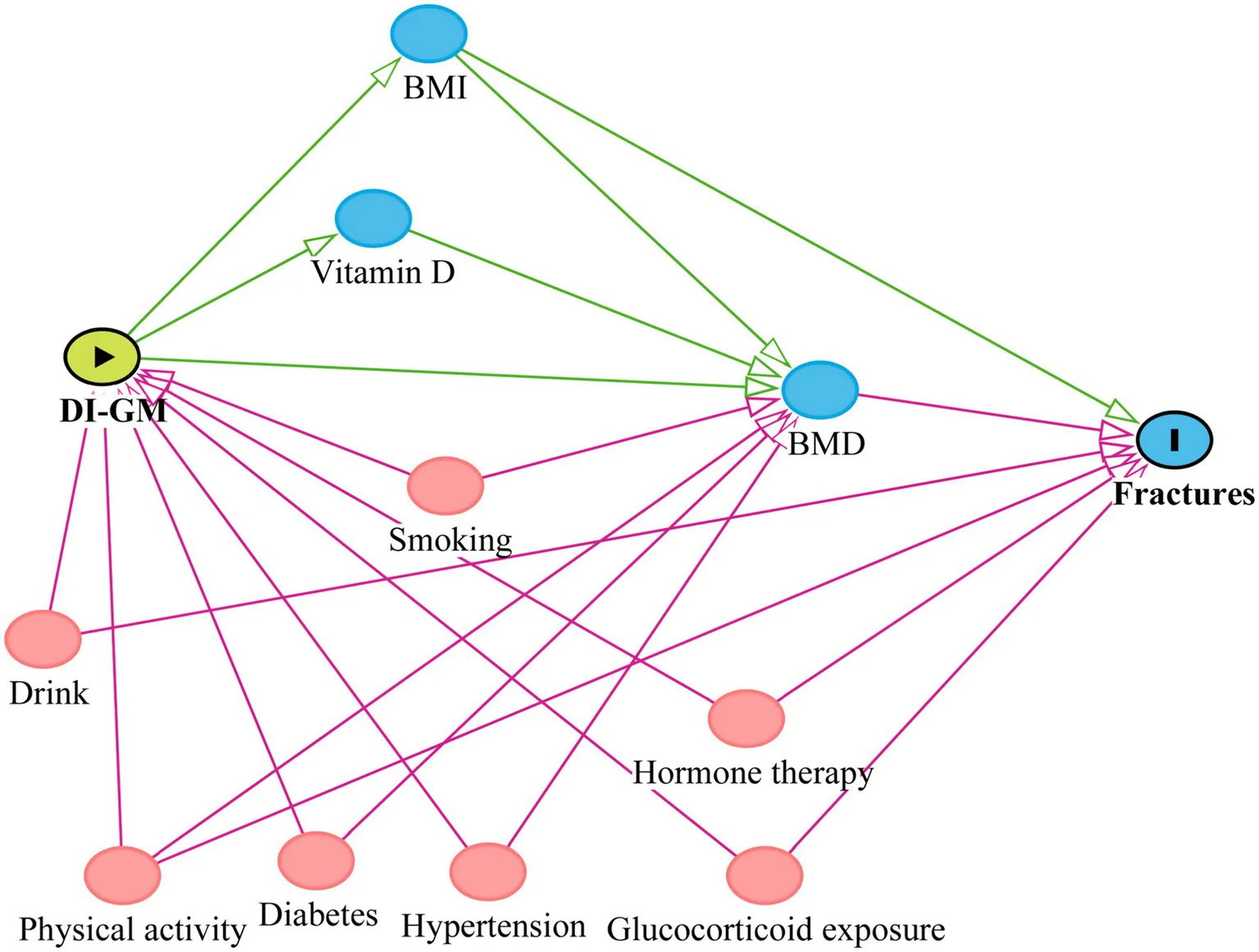
Directed acyclic graph (DAG) of the hypothesized relationships among DI-GM, BMD, fracture prevalence, and key covariates. The minimal sufficient adjustment set identified by the DAG included diabetes, alcohol consumption, glucocorticoid exposure, hormone therapy, hypertension, physical activity, and smoking. Age, race, and marital status were also treated as established confounders and adjusted for in all models but are not depicted in the DAG to maintain clarity. BMI and vitamin D are shown for completeness of hypothesized biological relationships but were not included in the adjustment set.

Race was categorized as non-Hispanic White, non-Hispanic Black, Mexican American, other Hispanic, and other races. Marital status was dichotomized into living with a partner (married or cohabiting) versus living alone (single, widowed, separated, or divorced). Education and poverty income ratio (PIR) were used for descriptive purposes only and were not included in the multivariable models. Educational attainment was classified into below high school, high school graduate, and college or postgraduate education. Family poverty status was stratified using PIR, with thresholds ≤ 1.30 indicating low income, 1.30–3.5 representing medium income, and >3.5 signifying high income (27). Smoking status was defined based on lifetime cigarette consumption, categorizing participants as never smokers (<100 cigarettes), former smokers (≥100 cigarettes but not currently smoking), and current smokers (≥100 cigarettes and currently smoking). Alcohol consumption was dichotomized as abstinent or consuming (having consumed at least 12 alcoholic drinks per year).

Hypertension diagnosis adhered to the American Heart Association/American College of Cardiology guidelines (28), incorporating self-reported data and meeting one of the following criteria: average systolic blood pressure ≥130 mmHg, average diastolic blood pressure ≥80 mmHg, previous medical diagnosis of hypertension, or current use of antihypertensive medication. Diabetes diagnosis followed the American Diabetes Association standards (29), including HbA1c > 6.5%, fasting glucose ≥7.0 mmol/L, random glucose ≥11.1 mmol/L, positive oral glucose tolerance test, previous diabetes diagnosis, or ongoing use of hypoglycemic drugs. Body mass index (BMI) was calculated as weight in kilograms divided by height in meters squared. Physical activity levels were classified according to metabolic equivalent task (MET) units, with <3 METs considered low intensity, 3–6 METs as moderate intensity, and >6 METs as high intensity (30, 31). Serum vitamin D concentrations were quantified using ultra-performance liquid chromatography–tandem mass spectrometry (UPLC-MS/MS), summing the concentrations of 25-hydroxyvitamin D3 [25(OH)D3], epi-25-hydroxyvitamin D3 [epi-25(OH)D3], and 25-hydroxyvitamin D2 [25(OH)D2].

Other potential confounders, such as total energy intake, calcium intake, protein intake, dietary supplementation, osteoporosis medication, fall history, and time since menopause, were not included in the models due to data unavailability or because they did not meet DAG criteria for confounding.

### 2.6 Statistical analysis

Descriptive statistics were used to summarize the data. Continuous variables following a normal distribution were presented as means ± standard deviations (SD), and those with skewed distributions were described using medians and interquartile ranges (IQR). Categorical variables were reported as frequencies and percentages. To examine the association between DI-GM and fracture prevalence, weighted logistic regression models were employed. Model 1 adjusted for age, race, and marital status. Model 2 further adjusted for alcohol consumption, smoking, hypertension, and diabetes. Model 3 additionally adjusted for physical activity, hormone therapy, and glucocorticoid exposure. The same model definitions were applied consistently across the main analysis, complete-case analysis, and multiple imputation analyses. Odds ratios (ORs) and 95% confidence intervals (CIs) were calculated for each model. Additionally, DI-GM scores were categorized into tertiles (Q1 ≤ 4, Q2 5–6, Q3 > 6) to assess dose–response associations, with *P* for trend calculated using the median value of each tertile.

Sensitivity analyses were conducted to evaluate the robustness of our findings. Subgroup analyses were performed based on age (50–64, ≥65 years), race (non-Hispanic White vs. other), smoking (never vs. former/current), alcohol consumption (no vs. yes), physical activity (low vs. moderate/vigorous), hypertension (no vs. yes), diabetes (no vs. yes), hormone therapy (no vs. yes), and glucocorticoid exposure (no vs. yes). Multiplicative interaction terms between DI-GM and each subgroup variable were added to the fully adjusted model, and *P* for interaction was obtained using a Wald test. Missing data were handled using multiple imputation by chained equations (MICE). We generated *m* = 20 imputed datasets and analyzed all 20 datasets using weighted logistic regression, with results combined using Rubin’s Rules. Continuous variables were imputed using predictive mean matching (PMM), binary variables using logistic regression (logreg), and multinomial variables using multinomial logistic regression (polyreg). The imputation model included all analytical variables, with the outcome and survey design variables as predictors. Femoral neck BMD and total spine BMD were not imputed and were not used as predictors for each other because of their high missing rates and because they were not included as covariates in the primary model. Convergence was assessed using trace plots (Supplementary File 2 Figure S1). Missing counts and rates are provided in Supplementary File 2 Table S1, and the predictor matrix is provided in Supplementary File 2 Table S2. Survey design variables were incorporated in both the imputation and analysis stages. Additionally, a complete-case analysis was performed by excluding all individuals with any missing data (*n* = 4,941) and reanalyzing the remaining dataset. To explore potential non-linear associations, restricted cubic splines (RCS) were utilized separately for any fracture and fragility fracture, with *P* for non-linearity reported.

Furthermore, exploratory mediation analysis was performed to evaluate the extent to which femoral neck and spinal BMD mediate the association between DI-GM and fracture prevalence. Femoral neck and lumbar spine BMD were selected as mediators because they are the skeletal sites recommended by the International Society for Clinical Densitometry (ISCD) for osteoporosis diagnosis. The analysis was conducted using the Baron and Kenny framework (32), with bootstrapping (5,000 resamples) to estimate the indirect effect and its 95%CI. The mediation model assumed sequential ignorability, i.e., that the mediator (BMD) and outcome (fracture) were not affected by unmeasured confounders given the observed covariates. Exposure-mediator interaction was not considered, as the cross-sectional design and the focus on total effect decomposition did not support the estimation of interactive effects. The analysis estimated both direct and indirect effects, and the proportion mediated was calculated as the indirect effect divided by the total effect.

Survey weights, strata (SDMVSTRA), and primary sampling units (SDMVPSU) were incorporated into all primary weighted logistic regression models using the svydesign() function in R’s survey package, with robust variance estimation. Subgroup analyses, restricted cubic spline analyses, and mediation analyses were conducted without applying survey weights. The rationale for this approach is as follows: subgroup analyses were primarily aimed at assessing effect modification (interaction) rather than producing nationally representative estimates within each stratum; restricted cubic spline analyses were exploratory, examining the functional form of the association; and mediation analyses using the mediation package with a binary outcome and continuous mediator can yield unstable estimates when survey weights are applied. This approach is consistent with prior NHANES-based studies examining effect modification and mediation (33, 34).

All statistical analyses were performed using R software (version 4.3.1, R Foundation for Statistical Computing, Vienna, Austria). A *p*-value < 0.05 was considered statistically significant.

### 2.7 Preliminary exploratory pilot analysis

To provide preliminary contextual support for the NHANES-based findings, we conducted a small exploratory pilot analysis in an independent cohort of 12 patients with osteoporosis recruited from the Outpatient Department of Sichuan Province Orthopedic Hospital between June 1, 2026 and June 15, 2026. The study protocol was reviewed and approved by the Ethics Committee of Sichuan Province Orthopedic Hospital (Approval No. KY2026-039-01). All participants provided written informed consent.

Eligible participants were women aged 50 years or older with a confirmed diagnosis of osteoporosis based on DXA-measured BMD (*T*-score ≤−2.5 at the lumbar spine or femoral neck, according to World Health Organization criteria). Patients with a history of metabolic bone diseases (other than osteoporosis), severe renal or hepatic dysfunction, or current use of medications known to affect bone metabolism (e.g., bisphosphonates, denosumab, teriparatide, or systemic glucocorticoids) were excluded. DI-GM scores were calculated using the same 13-component algorithm as in the NHANES analysis, based on dietary intake assessed by a validated semi-quantitative food frequency questionnaire (35). BMD at the lumbar spine (L1–L4) was measured using dual-energy X-ray absorptiometry (DXA). Normality of DI-GM scores and spinal BMD was assessed using the Shapiro–Wilk test. Pearson’s correlation coefficient was used to assess the association between DI-GM scores and spinal BMD, as both variables were approximately normally distributed. Given the small sample size, no adjustment for covariates was performed.

Given the exploratory nature and small sample size of this pilot analysis, all results should be interpreted with caution and considered as exploratory findings that require confirmation in larger, adequately powered prospective studies.

## 3 Results

### 3.1 Characteristics of study participants

The final analytical set comprised 6,318 women aged 50 years and older, with a mean age of 63.8 ± 9.6 years. Among these participants, 863 (13.7%) reported a history of fractures. Specifically, 311 women (36.0% of all fractures; 4.9% of the total cohort) had fragility fractures, defined as fractures resulting from a fall from standing height or less. Table 1 summarizes the demographic and clinical characteristics of the study population according to fracture. Notably, women with a history of fractures were generally older, more likely to be non-Hispanic white, living alone, current or former smokers, and had lower femoral neck and spinal bone densities compared to those without fractures. Women with fractures were also more likely to have used hormone therapy and to have been exposed to glucocorticoids. Serum vitamin D was slightly higher in women with fractures, whereas BMI did not differ between groups.

**Table 1:** Characteristics of the demographic grouped by fracture.

Variables	Total (*n* = 6,318)	Non-fracture (*n* = 5,455)	Fracture (*n* = 863)	*p*-value
Age (year)	63.8 ± 9.6	63.5 ± 9.5	66.1 ± 10.0	<0.001
Race (%)				**<0.001**
Non-Hispanic White	3,088 (75.55)	2,526 (73.81)	562 (85.38)	
Non-Hispanic Black	1,324 (10.30)	1,223 (11.19)	101 (5.26)	
Mexican American	865 (4.59)	777 (4.87)	88 (3.04)	
Other Race	1,041 (9.56)	929 (10.13)	112 (6.32)	
Marital status (%)				**0.019**
Coupled	3,133 (57.80)	2,731 (58.52)	402 (53.70)	
Singled	3,179 (42.20)	2,718 (41.48)	461 (46.30)	
PIR group (%)				0.169
≤1.3	1,637 (18.14)	1,393 (17.94)	244 (19.23)	
1.3–3.5	2,337 (38.37)	2009 (37.93)	328 (40.85)	
>3.5	1733 (43.49)	1,512 (44.13)	221 (39.92)	
Education (%)				0.561
<High school	1756 (16.97)	1,526 (16.89)	230 (17.47)	
High school	1,601 (27.63)	1,382 (27.98)	219 (25.67)	
>High school	2,950 (55.40)	2,538 (55.14)	412 (56.86)	
Smoking (%)				**0.017**
Never	3,854 (59.49)	3,391 (60.50)	463 (53.82)	
Former	1,606 (27.06)	1,345 (26.47)	261 (30.40)	
Current	855 (13.45)	716 (13.03)	139 (15.79)	
Drink (%)				0.119
No	2,405 (32.55)	2,104 (32.99)	301 (30.03)	
Yes	3,578 (67.45)	3,063 (67.01)	515 (69.97)	
PA intensity (%)				0.101
Low	2,372 (31.61)	2022 (30.80)	350 (36.19)	
Moderate	2,949 (49.27)	2,557 (49.77)	392 (46.45)	
High	997 (19.12)	876 (19.43)	121 (17.36)	
Hypertension (%)				0.365
No	2,179 (40.87)	1899 (41.19)	280 (39.07)	
Yes	4,139 (59.13)	3,556 (58.81)	583 (60.93)	
DM (%)				0.881
No	4,545 (78.71)	3,907 (78.75)	638 (78.47)	
Yes	1773 (21.29)	1,548 (21.25)	225 (21.53)	
Vitamin D (nmol/L)	75.6 ± 32.1	75.1 ± 31.6	78.6 ± 34.2	0.037
BMI (kg/m^2^)	29.4 ± 7.1	29.4 ± 7.1	29.5 ± 7.1	0.962
Femoral neck BMD (g/cm^2^)	0.719 ± 0.131	0.726 ± 0.128	0.680 ± 0.140	<0.001
Total spine BMD (g/cm^2^)	0.958 ± 0.154	0.962 ± 0.152	0.935 ± 0.167	0.014
Hormone therapy use, *n* (%)				**0.002**
No	3,838 (60.7)	3,357 (61.3)	481 (57.3)	
Yes	2092 (39.3)	1768 (38.7)	324 (42.7)	
Glucocorticoid exposure, n (%)				**<0.001**
No	4,828 (91.6)	4,193 (92.2)	635 (88.4)	
Yes	404 (8.4)	314 (7.8)	90 (11.6)	

Continuous variables were depicted using weighted means and standard deviations, whereas categorical variables were shown with raw frequencies and weighted percentages. PA, physical activity; DM, diabetes mellitus; BMI, body mass index; DI-GM, dietary index for gut microbiota; BMD, bone mineral density; SD, standard deviation.

### 3.2 Association between DI-GM and fracture prevalence

Table 2 presents the results of the weighted logistic regression models examining the association between DI-GM and fracture prevalence. In Model 1, adjusting for age, race, and marital status, each one-score increase in DI-GM was associated with 5% lower odds of prevalent fracture (OR = 0.95, 95% CI: 0.91–0.99, *p* = 0.018). After further adjustment for alcohol consumption, smoking, hypertension, and diabetes in Model 2, the inverse association remained significant (OR = 0.96, 95% CI: 0.92–1.00, *p* = 0.039). In Model 3, which additionally adjusted for physical activity, hormone therapy, and glucocorticoid exposure, the inverse association remained statistically significant (OR = 0.96, 95% CI: 0.92–0.99, *p* = 0.045).

**Table 2:** Weighted logistic regression analysis of the association between DI-GM and fracture prevalence (*n* = 6,318; events = 863).

Models	Crude model		Model-1		Model-2		Model-3	
	OR (95%)	*P*	OR (95%)	*P*	OR (95%)	*P*	OR (95%)	*P*
Scores	0.94 (0.88,1.01)	0.091	0.95 (0.91, 0.99)	0.018	0.96 (0.92, 1.00)	0.039	0.96 (0.92, 0.99)	0.045
Category								
Q1	Reference		Reference		Reference		Reference	
Q2	0.69 (0.53, 0.90)	0.007	0.98 (0.82, 1.18)	0.854	1.01 (0.83, 1.23)	0.895	1.01 (0.81, 1.25)	0.951
Q3	0.78 (0.60, 1.01)	0.058	0.69 (0.56, 0.86)	0.001	0.70 (0.55, 0.88)	0.002	0.66 (0.51, 0.85)	0.001
*P* for trend		0.052		0.003		0.008		0.006

DI-GM tertiles: Q1 [0, 4], Q2 [5, 6], Q3 [7, 13].

Model-1: age, race, marital status.

Model-2: Model-1 + alcohol, smoking, hypertension, diabetes.

Model-3: Model-2 + physical activity, HRT (estrogen), glucocorticoid.

To explore the dose–response relationship, DI-GM scores were categorized into three groups based on the distribution of scores in the study population: Q1 (≤4), Q2 (5–6), and Q3 (>6). Because DI-GM scores are discrete integers, exact tertiles were not achievable; therefore, we used the closest cut points that created three groups with distinct score ranges. The sample size and weighted proportion for each group were: Q1 (*n* = 2,642; weighted 42.1%), Q2 (*n* = 1,578; weighted 24.8%), and Q3 (n = 2,098; weighted 33.1%). In the fully adjusted model (Model 3), compared with the lowest tertile (Q1), the highest tertile (Q3) showed a significant inverse association (OR = 0.66, 95% CI: 0.51–0.85, *p* = 0.001), while the middle tertile (Q2) did not show a significant association (OR = 1.01, 95% CI: 0.81–1.25, *p* = 0.951). The trend test suggested a linear trend across tertiles (*P* for trend = 0.006).

### 3.3 Sensitivity analyses

Sensitivity analyses were conducted to evaluate the robustness of our findings. Subgroup analyses were exploratory. No statistically significant interactions were observed between DI-GM and age, race, smoking, alcohol consumption, physical activity, hypertension, diabetes, hormone therapy, or glucocorticoid exposure (all *P* for interaction > 0.05). Therefore, we did not identify any subgroup in which the association was significantly modified (see Figure 3).

**Figure 3:**
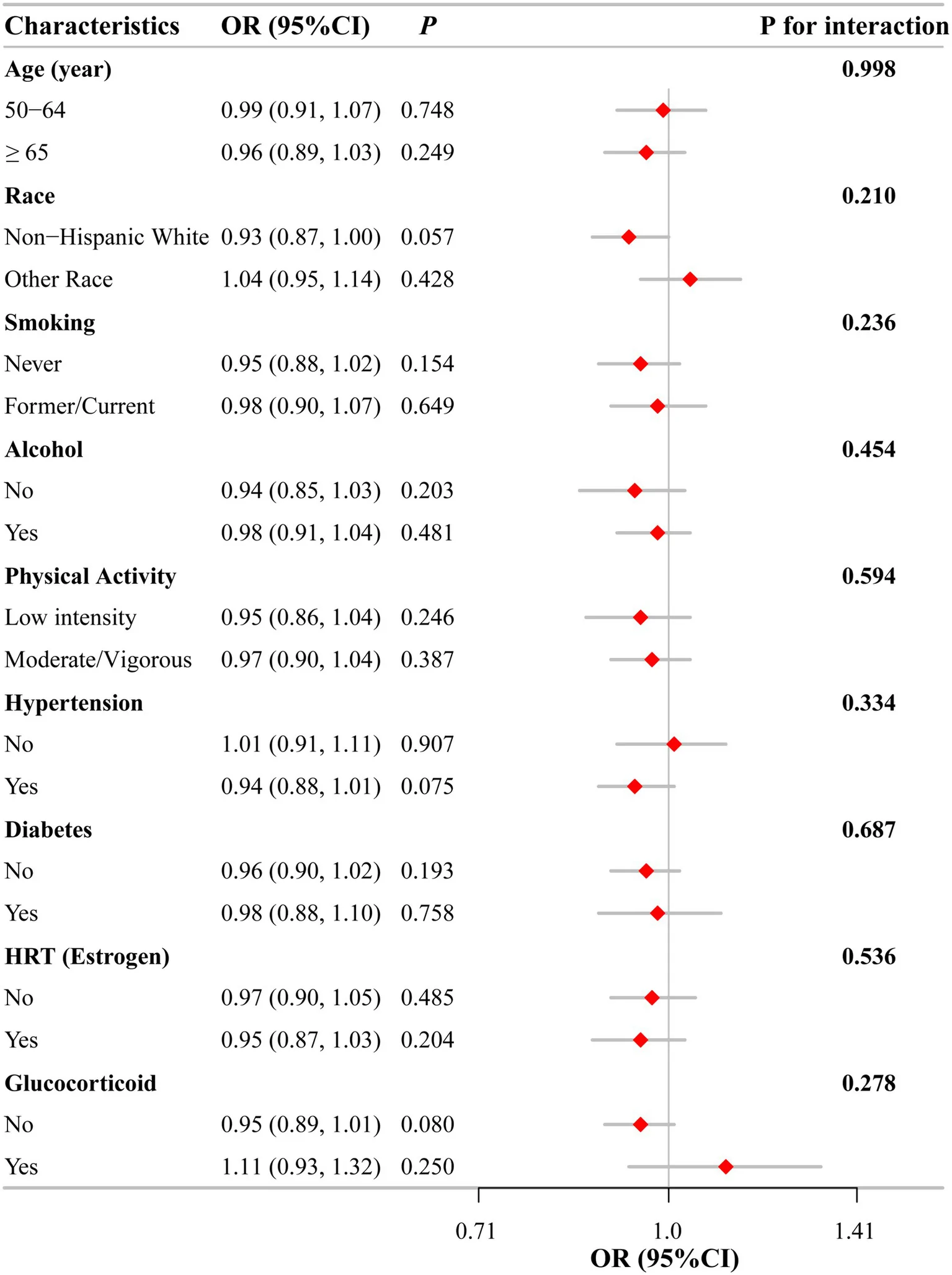
Subgroup analysis of the association between DI-GM and fracture prevalence. Unweighted logistic regression. DI-GM was modeled as a continuous variable. Models were adjusted for covariates in Model 3 except the subgroup variable itself. P for interaction was tested by adding a DI-GM × subgroup interaction term.

In the sensitivity analysis restricted to fragility fractures (defined as fractures caused by a fall from standing height or less, *n* = 311), the inverse association between DI-GM and fracture remained statistically significant. In the fully adjusted model, each one-score increase in DI-GM was associated with 7% lower odds of fragility fracture (OR = 0.93, 95% CI: 0.88–1.00, *p* = 0.035). When DI-GM was categorized into tertiles, compared with the lowest tertile (Q1), the middle tertile (Q2) showed a significant inverse association (OR = 0.61, 95% CI: 0.45–0.83, *p* = 0.001), and the highest tertile (Q3) also showed a significant inverse association (OR = 0.70, 95% CI: 0.53–0.93, *p* = 0.013). The trend test further indicated a significant trend across DI-GM tertiles (*P* for trend = 0.004) (Table 3).

**Table 3:** Weighted logistic regression analysis of the association between DI-GM and fragility fracture prevalence (*n* = 6,318; events = 311).

Models	Crude model		Model-1		Model-2		Model-3	
	OR (95%)	*P*	OR (95%)	*P*	OR (95%)	*P*	OR (95%)	*P*
Scores	0.97 (0.91, 1.02)	0.23	0.92 (0.87, 0.98)	0.013	0.94 (0.89, 1.00)	0.044	0.93 (0.88, 1.00)	0.035
Category								
Q1	Reference		Reference		Reference		Reference	
Q2	0.69 (0.53, 0.89)	0.004	0.60 (0.45, 0.80)	0.001	0.65 (0.49, 0.85)	0.002	0.61 (0.45, 0.83)	0.001
Q3	0.91 (0.72, 1.16)	0.446	0.75 (0.58, 0.97)	0.03	0.77 (0.60, 1.00)	0.046	0.70 (0.53, 0.93)	0.013
*P* for trend		0.153		0.006		0.013		0.004

DI-GM tertiles: Q1 [0, 4], Q2 [5, 6], Q3 [7, 13].

Model-1: age, race, marital status.

Model-2: Model-1 + alcohol, smoking, hypertension, diabetes.

Model-3: Model-2 + physical activity, HRT (estrogen), glucocorticoid.

In the complete-case analysis (*n* = 4,941), each 1-point increase in DI-GM was associated with lower odds of fracture prevalence in the fully adjusted model (OR = 0.96, 95% CI: 0.92–0.99, *p* = 0.045). Consistently, the highest DI-GM tertile showed a significant inverse association compared with the lowest tertile (OR = 0.66, 95% CI: 0.51–0.85, *p* = 0.001) (see Table 4).

**Table 4:** Weighted logistic regression analysis of the association between DI-GM and fracture after removal of all missing data (*n* = 4,941; events = 678).

Models	Crude model		Model-1		Model-2		Model-3	
	OR (95%)	*P*	OR (95%)	*P*	OR (95%)	*P*	OR (95%)	*P*
Scores	0.97 (0.96, 0.99)	0	0.96 (0.91, 1.00)	0.049	0.96 (0.91, 1.01)	0.08	0.96 (0.92, 0.99)	0.045
Category								
Q1	Reference		Reference		Reference		Reference	
Q2	0.93 (0.53, 1.64)	0.804	1.00 (0.81, 1.22)	0.97	1.00 (0.80, 1.25)	0.988	1.01 (0.81, 1.25)	0.951
Q3	0.96 (0.91, 1.00)	0.068	0.66 (0.51, 0.85)	0.001	0.67 (0.52, 0.86)	0.002	0.66 (0.51, 0.85)	0.001
*P* for trend		0.02		0.005		0.009		0.006

DI-GM tertiles: Q1 [0, 4], Q2 [5, 6], Q3 [7, 13].

Model-1: age, race, marital status.

Model-2: Model-1 + alcohol, smoking, hypertension, diabetes.

Model-3: Model-2 + physical activity, HRT (estrogen), glucocorticoid.

Pooled results from the 20 imputed datasets using Rubin’s Rules are presented in Table 5. The association between DI-GM and fracture prevalence remained statistically significant (OR = 0.95, 95% CI: 0.92–0.99, *p* = 0.025) (see Table 5).

**Table 5:** Pooled results from multiple imputation analysis for the association between DI-GM and fracture prevalence.

Models	Crude model		Model-1		Model-2		Model-3	
	OR (95%)	*P*	OR (95%)	*P*	OR (95%)	*P*	OR (95%)	*P*
Scores	0.98 (0.94, 1.01)	0.203	0.95 (0.91, 0.99)	0.013	0.95 (0.91, 0.99)	0.018	0.95 (0.92, 0.99)	0.025
Category								
Q1	Reference		Reference		Reference		Reference	
Q2	1.05 (0.88, 1.25)	0.59	0.98 (0.82, 1.18)	0.86	0.99 (0.82, 1.20)	0.914	1.00 (0.83, 1.21)	0.979
Q3	0.78 (0.64, 0.96)	0.016	0.69 (0.56, 0.85)	<0.001	0.69 (0.56, 0.85)	<0.001	0.69 (0.56, 0.85)	<0.001
*P* for trend		0.054		0.002		0.003		0.003

Multiple imputation was performed with *m* = 20 imputed datasets. Results were combined using Rubin’s Rules. Weighted logistic regression models were used. DI-GM tertiles: Q1 [0, 4], Q2 [5, 6], Q3 [7, 13].

Model-1: age, race, marital status.

Model-2: Model-1 + alcohol, smoking, hypertension, diabetes.

Model-3: Model-2 + physical activity, HRT (estrogen), glucocorticoid.

In the RCS analysis, the association between DI-GM and any fracture was approximately linear (*P* for non-linearity = 0.865), whereas the association with fragility fracture showed significant non-linearity (*P* for non-linearity = 0.012), with a reference point at a DI-GM score of 5 (see Figure 4). For fragility fracture, the RCS curve exhibited a U-shaped association, with the lowest odds around a DI-GM score of 5 and increased risk at both lower and higher scores. However, given the exploratory nature of this analysis and the limited number of fragility fracture events (*n* = 311), this finding should be interpreted with caution.

**Figure 4:**
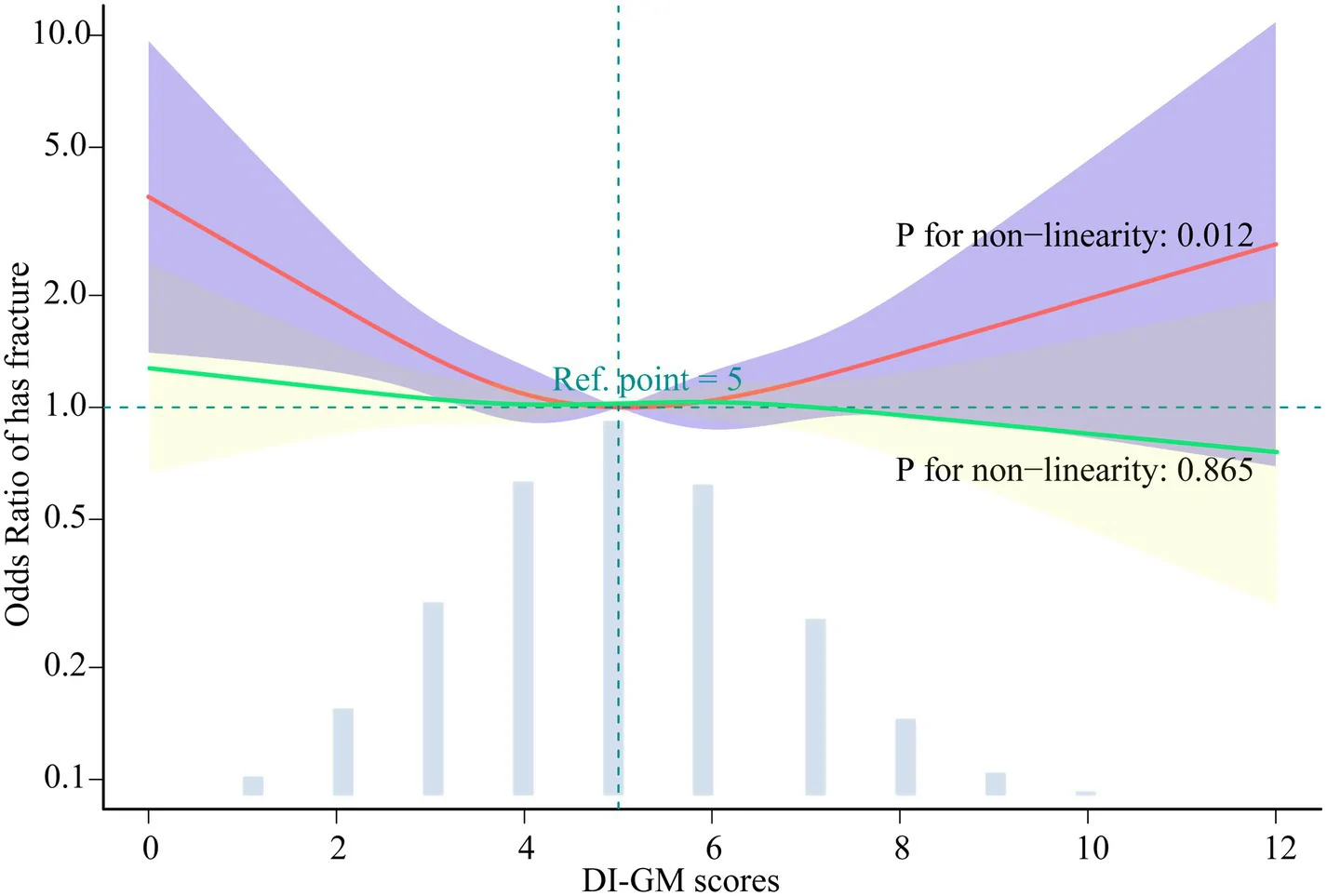
Restricted cubic spline analysis of the association between DI-GM scores and fracture prevalence. The green line represents any fracture; the red line represents fragility fracture. Unweighted restricted cubic spline analysis. DI-GM, dietary index for gut microbiota.

### 3.4 Mediation analysis

Exploratory mediation analysis was conducted to statistically decompose the association between DI-GM and fracture prevalence. For femoral neck BMD, the path from DI-GM to BMD was not statistically significant (*β* = 0.016, *p* = 0.162), whereas BMD was significantly associated with fracture (*β* = −0.234, *p* = 0.001). The indirect effect through femoral neck BMD was not statistically significant (*p* = 0.168). For spinal BMD, the path from DI-GM to BMD was not significant (*β* = 0.003, *p* = 0.791), while BMD was significantly associated with fracture (*β* = −0.161, *p* = 0.014). The indirect effect through spinal BMD was also not statistically significant (*p* = 0.800) (see Figure 5). These findings suggest that BMD did not significantly mediate the association between DI-GM and fracture prevalence in this exploratory analysis.

**Figure 5:**
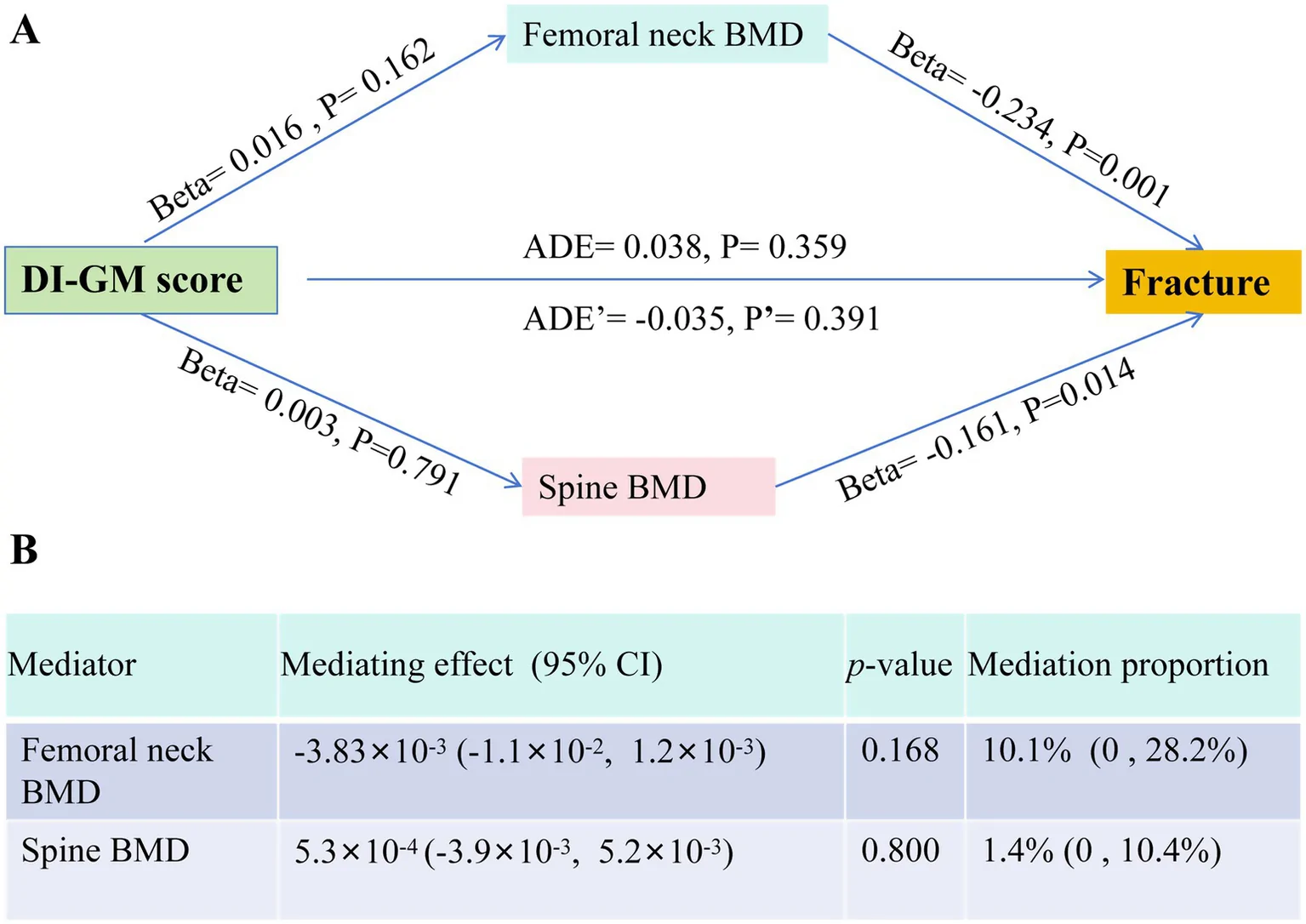
Exploratory statistical mediation model of femoral neck and spinal BMD. **(A)** Path coefficients and direct effects; **(B)** mediation effects and proportions. DI-GM, dietary index for gut microbiota; BMD, bone mineral density; ADE, average direct effect. Neither mediation proportion was statistically significant, and given the cross-sectional design, these mediation proportions should not be interpreted as causal pathway estimates.

### 3.5 Exploratory pilot analysis

In the exploratory pilot cohort (*n* = 12), the mean age was 64.9 ± 11.1 years, and the mean DI-GM score was 4.6 ± 1.4 (see Table 6). Considerable inter-individual variability was observed in age, DI-GM score, and spinal BMD (see Figure 6A). Correlation analysis demonstrated a significant positive association between DI-GM score and spinal BMD (*R* = 0.82, *p* = 0.0011), suggesting that a higher DI-GM score may be associated with better bone health status (see Figure 6B). In addition, representative imaging findings revealed apparent heterogeneity in skeletal manifestations among patients (see Figure 6C). These preliminary observations are consistent with the direction of the NHANES-based findings. However, given the small sample size and exploratory nature of this pilot analysis, these results should not be interpreted as confirmatory validation. Larger, adequately powered studies are needed to formally evaluate the DI-GM–BMD association in independent populations.

**Table 6:** Characteristics of the preliminary exploratory pilot cohort.

Variable	Total (*n* = 12)	Non-fracture history (*n* = 9)	Fracture history (*n* = 3)
Age (years)	64.9 ± 11.1	65.0 ± 12.0	64.7 ± 10.0
Marital status, *n* (%)			
Coupled	9 (75.0)	6 (66.7)	3 (100)
Single	3 (25.0)	3 (33.3)	0 (0)
Total spine BMD (g/cm^2^)	0.9 ± 0.2	1.0 ± 0.1	0.8 ± 0.2
Smoking, *n* (%)			
No	11 (91.7)	8 (88.9)	3 (100)
Yes	1 (8.3)	1 (11.1)	0 (0)
Alcohol consumption, *n* (%)			
No	10 (83.3)	7 (77.8)	3 (100)
Yes	2 (16.7)	2 (22.2)	0 (0)
Vitamin D (nmol/L)	53.5 ± 9.9	51.7 ± 10.0	59.2 ± 8.8
Physical activity, *n* (%)			
No	3 (25.0)	3 (33.3)	0 (0)
Yes	9 (75.0)	6 (66.7)	3 (100)
Hypertension, *n* (%)			
No	9 (75.0)	7 (77.8)	2 (66.7)
Yes	3 (25.0)	2 (22.2)	1 (33.3)
Diabetes, *n* (%)			
No	9 (75.0)	7 (77.8)	2 (66.7)
Yes	3 (25.0)	2 (22.2)	1 (33.3)
BMI (kg/m^2^)	25.8 ± 4.2	25.7 ± 4.2	26.2 ± 5.3
DI-GM score	4.6 ± 1.4	5.3 ± 1.6	4.3 ± 0.6

Data are presented as mean ± standard deviation (SD) for continuous variables and *n* (%) for categorical variables. Due to the small sample size of this pilot cohort, no statistical comparisons between groups were performed. Smoking was defined as having smoked cumulatively for 100 cigarettes or more during one‘s lifetime (current or former). Alcohol consumption was defined as having consumed alcoholic beverages in the past year. Physical activity was defined as engaging in moderate- or vigorous-intensity leisure-time physical activity for ≥30 min per session, ≥3 times per week, according to the Healthy China Action (2019–2030). Hypertension and diabetes were based on self-reported previous diagnosis by a healthcare professional. DI-GM, dietary index for gut microbiota; BMD, bone mineral density.

**Figure 6:**
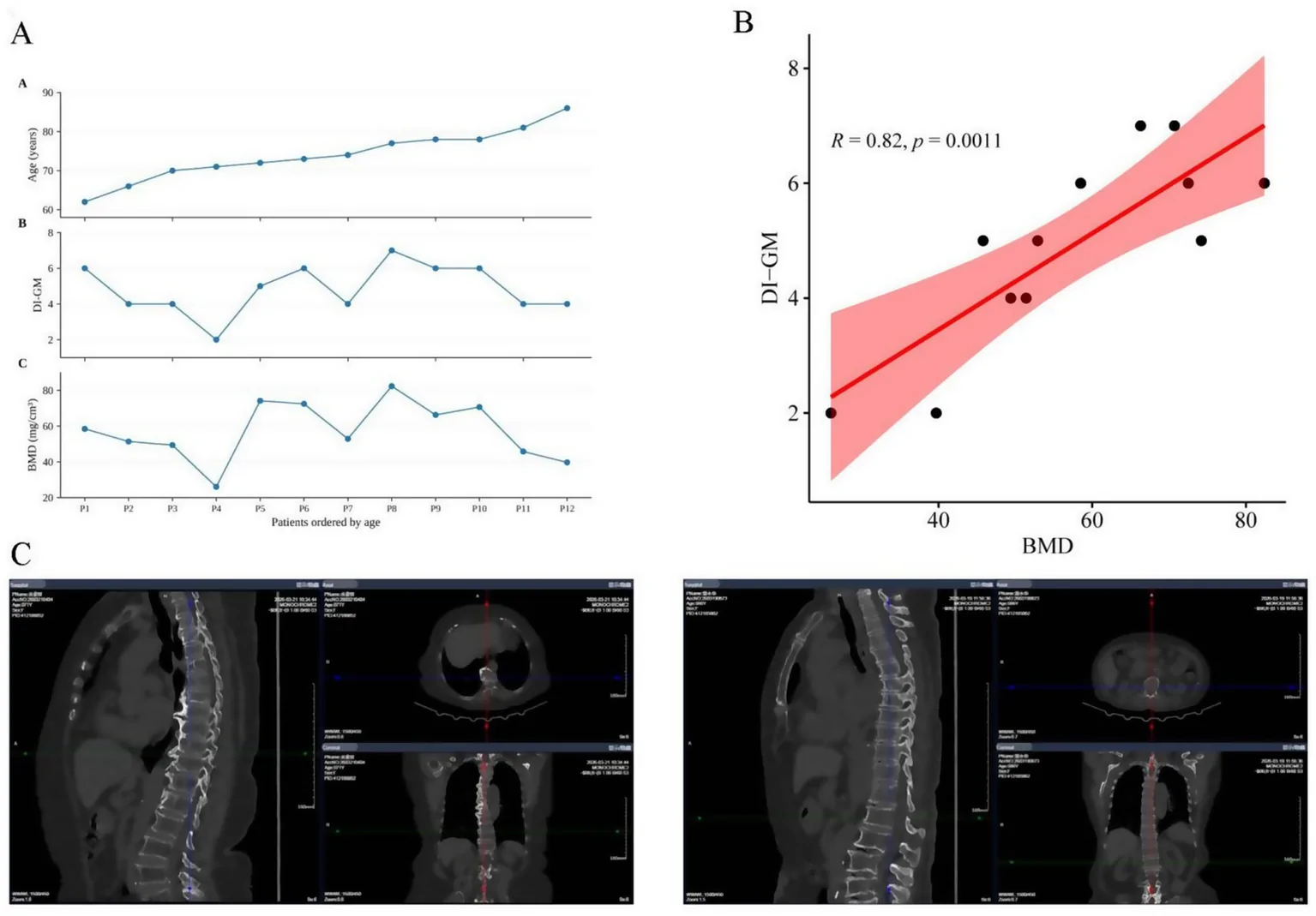
Preliminary exploratory pilot analysis in an osteoporosis cohort. **(A)** Basic information of patients; **(B)** correlation analysis results; **(C)** representative imaging.

## 4 Discussion

In this study, we used data from multiple cycles of NHANES to investigate the association between the DI-GM and fracture prevalence among women aged 50 years and older. We found that higher DI-GM scores were significantly associated with lower odds of both any fracture and fragility fracture, and this association was broadly consistent across multivariable adjustment, complete-case analysis, and multiple imputation. Exploratory mediation analysis did not show a statistically significant mediating role of femoral neck or lumbar spine BMD in the association between DI-GM and fracture prevalence. In addition, the exploratory pilot cohort showed a positive correlation between DI-GM scores and spinal BMD. Collectively, these findings demonstrate a significant cross-sectional association between dietary patterns characterized by higher DI-GM scores and lower fracture prevalence, as well as better bone health, in women aged 50 years and older.

Previous studies on diet and bone health have mainly focused on individual nutrients, specific food groups, or conventional dietary patterns, such as calcium, vitamin D, protein intake, the Mediterranean diet, the DASH diet, and the dietary inflammatory index (36–40). In contrast, DI-GM is a comprehensive dietary index based on literature-identified food components hypothesized to influence gut microbial health (11), but evidence regarding its association with fracture prevalence remains limited. Our findings are consistent with the emerging literature on DI-GM and bone health. The inverse association between DI-GM and fracture prevalence observed in our study aligns with the pooled analysis by Hidayat et al. (41). Similarly, Fang et al. (15) found that higher DI-GM scores were associated with lower odds of osteoporosis in postmenopausal women. Our study extends these findings by examining femoral neck and spinal BMD as potential mediators; however, the mediation effects were not statistically significant. This contrasts with Li et al., who identified caffeine and biological age as mediators of the DI-GM–osteoporosis association, explaining 20.33% and 4.73–4.55% of the effect, respectively (16). Our study also extends previous work by focusing specifically on fragility fractures and by using a DAG-based covariate selection strategy to minimize confounding.

From a mechanistic perspective, the association between DI-GM and bone health may be partly related to gut microbiota and its metabolic functions. Diets rich in dietary fiber, whole grains, legumes, and fermented foods may help maintain gut microbial diversity and promote the production of beneficial metabolites, such as short-chain fatty acids (42, 43). These metabolites may participate in bone metabolism by improving intestinal barrier function, modulating immune and inflammatory responses, enhancing mineral absorption, and regulating the activity of osteoblasts and osteoclasts (44, 45). Notably, femoral neck and lumbar spine BMD did not significantly mediate the association between DI-GM and fracture prevalence, suggesting that pathways other than BMD—such as bone quality (46), muscle function (45), fall risk (47), and chronic inflammation (48)—may contribute to this relationship. The present findings have important public health implications. Women aged 50 years and older are at elevated risk for osteoporosis and fractures, and fractures are often associated with long-term functional impairment, increased healthcare burden, and elevated mortality risk. Dietary modification is non-invasive, low-cost, sustainable, and broadly applicable at the population level. If the association between DI-GM and fracture prevalence is further confirmed in future studies, promoting the intake of whole grains, legumes, fiber-rich vegetables, fruits, and fermented foods, while reducing the consumption of processed meat, refined grains, and high-fat foods, may serve as a useful adjunctive strategy for fracture prevalence management in this population.

In addition to the NHANES-based primary analysis, we conducted a small exploratory pilot analysis in an independent osteoporosis cohort (n = 12). In this small exploratory pilot cohort, DI-GM scores were positively correlated with spinal BMD. However, given the small sample size, the examination of BMD rather than fracture as the outcome, and the lack of adjustment for confounders, these observations should be considered exploratory and hypothesis-generating. Future studies with larger sample sizes, prospective designs, and fracture as the primary outcome are warranted to formally validate the association between DI-GM and fracture prevalence in independent populations.

A major strength of this study is the use of nationally representative NHANES data, together with complex survey weighting, multivariable adjustment, sensitivity analyses, and exploratory mediation analysis, which collectively enhance the robustness of the findings. Several limitations should be acknowledged. First, because NHANES is cross-sectional, causal inference cannot be established, and reverse causation cannot be fully excluded. Second, fracture history and trauma mechanisms were self-reported, which may introduce recall bias; trauma mechanism data were also only available for fractures occurring at age 50 years or older. Third, dietary intake was assessed using 24-h recalls, which are subject to measurement error and may not fully capture long-term habitual dietary patterns; future studies using repeated recalls or food frequency questionnaires would improve exposure estimation. Fourth, although multiple covariates were adjusted for, residual confounding may still exist, including calcium or vitamin D supplementation, fall history, muscle mass, bone turnover markers, and gut microbiota composition. Fifth, the mediation analysis assumed sequential ignorability, which cannot be fully verified in cross-sectional data; the mediation proportions should therefore be interpreted as exploratory statistical decompositions rather than causal pathway estimates. Sixth, the exploratory pilot analysis had a small sample size, examined BMD rather than fracture as the outcome, and lacked adjustment for confounders; these findings should be considered hypothesis-generating. Finally, gut microbiota was not directly measured, and future prospective cohort studies and intervention trials integrating microbiota sequencing, metabolomics, inflammatory markers, and bone turnover markers are warranted to clarify causality and mechanisms.

## 5 Conclusion

In conclusion, this cross-sectional study highlights a significant inverse association between dietary patterns characterized by higher DI-GM scores and fracture prevalence among women aged 50 years and older. Exploratory mediation analysis did not show a statistically significant mediating role of femoral neck or spinal BMD, suggesting that other pathways may underlie the association between dietary patterns and skeletal health. Encouraging healthier dietary habits may be a promising strategy for fracture prevalence stratification and management, although future longitudinal studies are needed to establish temporality and causality. The overall research strategy and key conclusions of this study are summarized in Graphical abstract.

## Data Availability

The raw data supporting the conclusions of this article will be made available by the authors, without undue reservation.

## Footnotes

1
https://wwwn.cdc.gov/nchs/nhanes/Default.aspx
