# Supplementary material for: Association between the dietary index for gut microbiota and fracture prevalence among women aged 50 years and older: a NHANES-based study with a preliminary exploratory pilot analysis
Source: Front Public Health. 2026 Oct 6;14:1933908. doi: 10.3389/fpubh.2026.1933908 (PMC13680436; doi:10.3389/fpubh.2026.1933908)
Supplement: Supplementary file 1 [file supplementary_file_1.docx]

\# ============================================================================

\# NHANES 饮食数据下载 + DI-GM 计算（5 周期，Day 1+Day 2 两天平均）

\# 周期：2005-2006, 2007-2008, 2009-2010, 2013-2014, 2017-2018

\# 饮食日：Day 1 + Day 2 平均（对应权重 WTDR2D）

\# ============================================================================

\# DI-GM 13 个成分（NHANES 中排除绿茶）：

\#	有益（9项）：发酵乳制品、鹰嘴豆、大豆、全谷物、膳食纤维、蔓越莓、

\#	牛油果、西兰花、咖啡

\#	有害（4项）：红肉、加工肉、精制谷物、高脂饮食（≥40%总能量来自脂肪）

\# 评分：除高脂饮食用固定阈值外，其余按性别特异性中位数评分

\# ============================================================================

suppressPackageStartupMessages({

library(haven)

library(dplyr)

library(tidyr)

library(stringr)

library(writexl)

})

setwd("E:\\1.文章\\2026年\\5.肠菌指数\\肠菌--返修\\DIGM计算")

dir.create("nhanes_diet_data", showWarnings = FALSE)

\# ============================================================================

\# 第一部分：下载 NHANES 饮食数据（5 个周期，Day 1 + Day 2）

\# ============================================================================

\# 每个周期下载 6 个文件：

\#	DR1TOT_*.xpt — Day 1 总营养素

\#	DR2TOT_*.xpt — Day 2 总营养素

\#	DR1IFF_*.xpt — Day 1 单个食物

\#	DR2IFF_*.xpt — Day 2 单个食物

\#	DEMO_*.xpt	— 人口学

\#	DRXFCD_*.xpt — FNDDS 食物描述

\# ============================================================================

cat("============================================================\n")

cat(" 第一部分：下载 NHANES 饮食数据（5 周期，Day 1+2）\n")

cat("============================================================\n")

cycles <- data.frame(

label = c("2005-2006", "2007-2008", "2009-2010", "2013-2014", "2017-2018"),

suffix = c("D", "E", "F", "H", "J"),

year	= c("2005", "2007", "2009", "2013", "2017"),

stringsAsFactors = FALSE

)

base_url <- "https://wwwn.cdc.gov/Nchs/Data/Nhanes/Public"

download_nhanes_file <- function(filename, year, dest_dir = "nhanes_diet_data") {

url <- sprintf("%s/%s/DataFiles/%s", base_url, year, filename)

dest <- file.path(dest_dir, filename)

if (file.exists(dest)) {

cat(sprintf(" [已存在] %s\n", filename))

return(TRUE)

}

cat(sprintf(" [下载中] %s ... ", filename))

result <- tryCatch({

download.file(url, dest, mode = "wb", quiet = TRUE)

cat("完成\n")

TRUE

}, error = function(e) {

cat(sprintf("失败 (%s)\n", e$message))

cat(sprintf("	请手动从 %s 下载并放入 %s/\n", url, dest_dir))

FALSE

})

return(result)

}

all_downloads_ok <- TRUE

for (i in seq_len(nrow(cycles))) {

cyc <- cycles[i, ]

cat(sprintf("\n--- 周期 %s（后缀 %s）---\n", cyc$label, cyc$suffix))

files <- c(

sprintf("DR1TOT_%s.xpt", cyc$suffix),

sprintf("DR2TOT_%s.xpt", cyc$suffix),

sprintf("DR1IFF_%s.xpt", cyc$suffix),

sprintf("DR2IFF_%s.xpt", cyc$suffix),

sprintf("DEMO_%s.xpt", cyc$suffix),

sprintf("DRXFCD_%s.xpt", cyc$suffix)

)

for (f in files) {

ok <- download_nhanes_file(f, cyc$year)

if (!ok) all_downloads_ok <- FALSE

}

}

if (!all_downloads_ok) {

stop("\n部分文件下载失败，请手动下载后重新运行。")

}

cat("\n=== 全部下载完成 ===\n")

\# ============================================================================

\# 第二部分：计算 DI-GM（Day 1 + Day 2 两天平均）

\# ============================================================================

\# ----------------------------------------------------------------------------

\## 2.1 安全字符串处理函数（处理编码问题）

\# ----------------------------------------------------------------------------

safe_tolower <- function(x) {

x <- as.character(x)

out <- iconv(x, from = "latin1", to = "UTF-8", sub = "")

na_idx <- is.na(out)

if (any(na_idx)) {

out[na_idx] <- iconv(x[na_idx], from = "", to = "UTF-8", sub = "")

}

out[is.na(out)] <- ""

suppressWarnings(tolower(out))

}

\# ----------------------------------------------------------------------------

\## 2.2 加载并合并 5 个周期的数据（Day 1 + Day 2）

\# ----------------------------------------------------------------------------

cat("\n--- 加载数据 ---\n")

all_nutrient <- list()

all_food	<- list()

all_drxfcd	<- list()

for (i in seq_len(nrow(cycles))) {

cyc <- cycles[i, ]

cat(sprintf("加载 %s ... ", cyc$label))

\# --- Day 1 总营养素 ---

d1tot <- read_xpt(file.path("nhanes_diet_data",

sprintf("DR1TOT_%s.xpt", cyc$suffix)))

d1tot <- d1tot %>%

select(SEQN, DR1TKCAL, DR1TTFAT, DR1TFIBE, WTDRD1, DR1DRSTZ) %>%

filter(DR1DRSTZ == 1)	# 只保留 Day 1 可靠的受试者

\# --- Day 2 总营养素 ---

d2tot <- read_xpt(file.path("nhanes_diet_data",

sprintf("DR2TOT_%s.xpt", cyc$suffix)))

d2tot <- d2tot %>%

select(SEQN, DR2TKCAL, DR2TTFAT, DR2TFIBE, WTDR2D, DR2DRSTZ) %>%

filter(DR2DRSTZ == 1)	# 只保留 Day 2 可靠的受试者

\# 合并：保留所有 Day 1 可靠的受试者（left_join），

\# Day 2 缺失或不可靠的受试者，用 Day 1 数据填充 Day 2

tot <- d1tot %>%

left_join(d2tot, by = "SEQN") %>%

mutate(

cycle = cyc$label,

\# Day 2 缺失时用 Day 1 填充

DR2TKCAL = ifelse(is.na(DR2TKCAL), DR1TKCAL, DR2TKCAL),

DR2TTFAT = ifelse(is.na(DR2TTFAT), DR1TTFAT, DR2TTFAT),

DR2TFIBE = ifelse(is.na(DR2TFIBE), DR1TFIBE, DR2TFIBE),

\# 两天平均（Day 2 缺失时，平均 = Day 1 的值）

TKCAL = (DR1TKCAL + DR2TKCAL) / 2,

TTFAT = (DR1TTFAT + DR2TTFAT) / 2,

TFIBE = (DR1TFIBE + DR2TFIBE) / 2,

\# 权重：有两天可靠数据用 WTDR2D，只有 Day 1 用 WTDRD1

WTDR = ifelse(is.na(WTDR2D), WTDRD1, WTDR2D),

\# 标记是否有两天可靠数据

has_day2 = ifelse(is.na(WTDR2D), 0, 1)

) %>%

filter(TKCAL > 0) %>%

select(SEQN, cycle, TKCAL, TTFAT, TFIBE, WTDR, has_day2)

\# --- Day 1 单个食物 ---

d1iff <- read_xpt(file.path("nhanes_diet_data",

sprintf("DR1IFF_%s.xpt", cyc$suffix)))

d1iff <- d1iff %>%

select(SEQN, DR1IFDCD, DR1IGRMS) %>%

rename(FDCD = DR1IFDCD, GRMS = DR1IGRMS) %>%

mutate(day = 1)

\# --- Day 2 单个食物 ---

d2iff <- read_xpt(file.path("nhanes_diet_data",

sprintf("DR2IFF_%s.xpt", cyc$suffix)))

d2iff <- d2iff %>%

select(SEQN, DR2IFDCD, DR2IGRMS) %>%

rename(FDCD = DR2IFDCD, GRMS = DR2IGRMS) %>%

mutate(day = 2)

\# 只保留在 tot 中的受试者（Day 1 可靠）

d1iff <- d1iff %>% filter(SEQN %in% tot$SEQN)

d2iff <- d2iff %>% filter(SEQN %in% tot$SEQN)

\# 对于只有 Day 1 食物数据的受试者，复制 Day 1 条目作为 Day 2

only_day1_seqn <- setdiff(unique(d1iff$SEQN), unique(d2iff$SEQN))

d2iff_fill <- d1iff %>%

filter(SEQN %in% only_day1_seqn) %>%

mutate(day = 2)

\# 合并实际 Day 2 + 填充的 Day 2

d2iff_combined <- bind_rows(d2iff, d2iff_fill)

\# 合并两天食物，按 SEQN + food code 求两天平均

\# 注意：必须用 sum/2 而非 mean，因为某食物若只在一天吃了，另一天摄入量为 0

\#	mean 只有一个值会得到那一天的克数（偏高），sum/2 才是正确的两天平均

\# （对于只有 Day 1 的受试者，Day 2 = Day 1 填充，sum/2 = Day 1 的值）

iff <- bind_rows(d1iff, d2iff_combined) %>%

group_by(SEQN, FDCD) %>%

summarise(GRMS = sum(GRMS, na.rm = TRUE) / 2, .groups = "drop") %>%

mutate(cycle = cyc$label)

\# --- 人口学 ---

dem <- read_xpt(file.path("nhanes_diet_data",

sprintf("DEMO_%s.xpt", cyc$suffix)))

dem <- dem %>% select(SEQN, RIAGENDR, RIDAGEYR)

\# --- FNDDS 食物描述 ---

fcd <- read_xpt(file.path("nhanes_diet_data",

sprintf("DRXFCD_%s.xpt", cyc$suffix)))

fcd <- fcd %>%

mutate(

FDCD = as.numeric(DRXFDCD),

food_name = safe_tolower(DRXFCSD)

) %>%

select(FDCD, food_name)

\# 合并人口学

tot <- tot %>% left_join(dem, by = "SEQN")

all_nutrient[[i]] <- tot

all_food[[i]]	<- iff

all_drxfcd[[i]]	<- fcd

cat(sprintf("%d 人（Day1可靠，Day2缺失用Day1填充）, %d 条食物记录\n", nrow(tot), nrow(iff)))

}

\# 合并所有周期

nutrient_all <- bind_rows(all_nutrient)

food_all	<- bind_rows(all_food)

drxfcd_all	<- bind_rows(all_drxfcd) %>%

distinct(FDCD, .keep_all = TRUE) %>%

mutate(food_name = suppressWarnings(

iconv(food_name, from = "UTF-8", to = "UTF-8", sub = "")

))

cat(sprintf("\n合并后：%d 人（5 周期，Day1可靠，Day2缺失用Day1填充），%d 条食物记录\n",

nrow(nutrient_all), nrow(food_all)))

\# ----------------------------------------------------------------------------

\## 2.3 数据清洗：仅成年人

\# ----------------------------------------------------------------------------

cat("\n--- 数据清洗 ---\n")

nutrient_clean <- nutrient_all %>%

filter(!is.na(RIAGENDR), RIDAGEYR >= 18)

cat(sprintf("成年人：%d 人（原始 %d 人）\n",

nrow(nutrient_clean), nrow(nutrient_all)))

food_clean <- food_all %>%

filter(SEQN %in% nutrient_clean$SEQN)

cat(sprintf("食物记录：%d 条\n", nrow(food_clean)))

\# ----------------------------------------------------------------------------

\## 2.4 食物分类（food code 前缀 + FNDDS 名称关键词，改进版）

\# ----------------------------------------------------------------------------

cat("\n--- 食物分类 ---\n")

food_clean <- food_clean %>%

mutate(

FDCD_char = as.character(FDCD),

code1 = str_sub(FDCD_char, 1, 1),

code2 = str_sub(FDCD_char, 1, 2)

) %>%

left_join(drxfcd_all, by = "FDCD") %>%

mutate(food_name = ifelse(is.na(food_name), "", food_name))

\# --- 2.4.1 发酵乳制品（Fermented dairy）---

\# food code 14 = Yogurt；名称含 yogurt/kefir/fermented milk/yoghurt

\# 注意：两天平均后零摄入率会降低

food_clean <- food_clean %>%

mutate(

fermented_dairy = ifelse(

code2 == "14" | str_detect(food_name, "yogurt|kefir|fermented milk|yoghurt|sour cream"),

GRMS, 0)

)

\# --- 2.4.2 鹰嘴豆（Chickpeas）---

food_clean <- food_clean %>%

mutate(

chickpeas = ifelse(

str_detect(food_name, "chickpea|garbanzo|hummus"),

GRMS, 0)

)

\# --- 2.4.3 大豆（Soybeans）---

food_clean <- food_clean %>%

mutate(

soybeans = ifelse(

str_detect(food_name, "soy|tofu|edamame|soya|miso|tempeh|soybean"),

GRMS, 0)

)

\# --- 2.4.4 全谷物（Whole grains）—— 改进版关键词 ---

\# 关键改进：

\#	(1) 增加 "brown.*rice|rice.*brown" 匹配不同词序

\#	(2) 增加 "bran|graham|multigrain|7 grain|nine grain|steel cut|cracked wheat"

\#	(3) 增加 "whole" 单独匹配（限定在谷物组 code1==5 内，避免匹配 whole milk）

\#	(4) 增加 "oat" 相关更全面匹配

food_clean <- food_clean %>%

mutate(

whole_grains = ifelse(

code1 == "5" & str_detect(food_name,

"whole grain|whole wheat|whole-grain|wholewheat|brown.*rice|rice.*brown|wild rice|oatmeal|oats|oat bran|rolled oat|steel cut oat|instant oat|quinoa|barley|bulgur|buckwheat|amaranth|millet|sorghum|popcorn|rye|whole rye|bran|wheat bran|corn bran|graham|multigrain|multi-grain|7 grain|nine grain|12 grain|cracked wheat|couscous.*whole|whole.*couscous"),

GRMS, 0)

)

\# --- 2.4.5 蔓越莓（Cranberries）---

food_clean <- food_clean %>%

mutate(

cranberries = ifelse(

str_detect(food_name, "cranberry"),

GRMS, 0)

)

\# --- 2.4.6 牛油果（Avocados）---

food_clean <- food_clean %>%

mutate(

avocados = ifelse(

str_detect(food_name, "avocado|guacamole"),

GRMS, 0)

)

\# --- 2.4.7 西兰花（Broccoli）---

food_clean <- food_clean %>%

mutate(

broccoli = ifelse(

str_detect(food_name, "broccoli"),

GRMS, 0)

)

\# --- 2.4.8 咖啡（Coffee）---

food_clean <- food_clean %>%

mutate(

coffee = ifelse(

code2 == "93" | str_detect(food_name, "coffee|espresso|cappuccino|latte|mocha|macchiato"),

GRMS, 0)

)

\# --- 2.4.9 红肉（Red meat）---

food_clean <- food_clean %>%

mutate(

red_meat = ifelse(

code2 %in% c("21", "22", "23", "24", "81", "82") |

str_detect(food_name,

"beef|pork|veal|lamb|hamburger|steak|chop|roast|ribs|bacon|ham|sausage|hot dog|frankfurter|pepperoni|salami|bologna|pastrami|corned beef|pancetta|prosciutto|meatloaf|meat ball|meatball"),

GRMS, 0)

)

\# --- 2.4.10 加工肉（Processed meat）---

food_clean <- food_clean %>%

mutate(

processed_meat = ifelse(

code2 %in% c("27", "28") |

str_detect(food_name,

"bacon|sausage|hot dog|hotdog|ham|salami|bologna|pepperoni|pastrami|corned beef|frankfurter|bratwurst|kielbasa|luncheon meat|cured meat|smoked meat|dried meat|jerky|pancetta|prosciutto|guanciale|lardons|spam|vienna sausage"),

GRMS, 0)

)

\# --- 2.4.11 精制谷物（Refined grains）---

\# 谷物总量 − 全谷物

food_clean <- food_clean %>%

mutate(

all_grains = ifelse(code1 == "5", GRMS, 0),

refined_grains = pmax(all_grains - whole_grains, 0)

)

\# 检查各类食物总摄入量

cat("\n各类食物总摄入量（克，全部样本汇总）：\n")

food_totals <- food_clean %>%

summarise(across(c(fermented_dairy, chickpeas, soybeans, whole_grains,

cranberries, avocados, broccoli, coffee,

red_meat, processed_meat, refined_grains),

~ sum(.x, na.rm = TRUE))) %>%

round(1)

print(t(food_totals))

\# ----------------------------------------------------------------------------

\## 2.5 按受试者汇总

\# ----------------------------------------------------------------------------

cat("\n--- 按受试者汇总 ---\n")

food_by_subject <- food_clean %>%

group_by(SEQN, cycle) %>%

summarise(

fermented_dairy = sum(fermented_dairy, na.rm = TRUE),

chickpeas	= sum(chickpeas, na.rm = TRUE),

soybeans	= sum(soybeans, na.rm = TRUE),

whole_grains	= sum(whole_grains, na.rm = TRUE),

cranberries	= sum(cranberries, na.rm = TRUE),

avocados	= sum(avocados, na.rm = TRUE),

broccoli	= sum(broccoli, na.rm = TRUE),

coffee	= sum(coffee, na.rm = TRUE),

red_meat	= sum(red_meat, na.rm = TRUE),

processed_meat = sum(processed_meat, na.rm = TRUE),

refined_grains = sum(refined_grains, na.rm = TRUE),

.groups = "drop"

)

di_gm_data <- nutrient_clean %>%

left_join(food_by_subject, by = c("SEQN", "cycle")) %>%

mutate(across(c(fermented_dairy, chickpeas, soybeans, whole_grains,

cranberries, avocados, broccoli, coffee,

red_meat, processed_meat, refined_grains),

~ ifelse(is.na(.x), 0, .x)))

\# 脂肪供能比

di_gm_data <- di_gm_data %>%

mutate(

fat_energy_pct = TTFAT * 9 / TKCAL * 100,

high_fat_diet = ifelse(fat_energy_pct >= 40, 1, 0)

)

cat(sprintf("合并后样本：%d 名成年人\n", nrow(di_gm_data)))

cat(sprintf("高脂饮食（脂肪供能≥40%%）比例：%.1f%%\n",

mean(di_gm_data$high_fat_diet, na.rm = TRUE) * 100))

\# 检查各成分中位数（关键：确认中位数不为 0）

cat("\n=== 各成分中位数检查（两天平均后）===\n")

components <- c("fermented_dairy","chickpeas","soybeans","whole_grains",

"TFIBE","cranberries","avocados","broccoli","coffee",

"red_meat","processed_meat","refined_grains")

for (c in components) {

med <- median(di_gm_data[[c]], na.rm = TRUE)

pct_zero <- mean(di_gm_data[[c]] == 0, na.rm = TRUE) * 100

cat(sprintf(" %-20s 中位数=%8.2f 零摄入=%.1f%%\n", c, med, pct_zero))

}

\# ----------------------------------------------------------------------------

\## 2.6 性别特异性中位数（合并 5 周期总样本）

\# ----------------------------------------------------------------------------

cat("\n--- 计算性别特异性中位数 ---\n")

beneficial_components <- c("fermented_dairy", "chickpeas", "soybeans",

"whole_grains", "TFIBE", "cranberries",

"avocados", "broccoli", "coffee")

detrimental_components <- c("red_meat", "processed_meat", "refined_grains")

medians <- di_gm_data %>%

group_by(RIAGENDR) %>%

summarise(

across(c(all_of(beneficial_components), all_of(detrimental_components)),

~ median(.x, na.rm = TRUE), .names = "median_{.col}"),

.groups = "drop"

)

cat("\n性别特异性中位数：\n")

print(t(medians))

di_gm_data <- di_gm_data %>%

left_join(medians, by = "RIAGENDR")

\# ----------------------------------------------------------------------------

\## 2.7 DI-GM 评分

\# ----------------------------------------------------------------------------

cat("\n--- DI-GM 评分 ---\n")

di_gm_data <- di_gm_data %>%

mutate(

\# 有益成分：> 中位数 → 1 分（严格大于，原始论文 "above the median"）

\# 注意：必须用 > 而非 >=，否则中位数为0时所有人都得分（100%），

\#	导致鹰嘴豆、大豆、蔓越莓、牛油果、西兰花等稀有食物失去区分度

score_fermented_dairy = ifelse(fermented_dairy > median_fermented_dairy, 1, 0),

score_chickpeas	= ifelse(chickpeas > median_chickpeas, 1, 0),

score_soybeans	= ifelse(soybeans > median_soybeans, 1, 0),

score_whole_grains	= ifelse(whole_grains > median_whole_grains, 1, 0),

score_fiber	= ifelse(TFIBE > median_TFIBE, 1, 0),

score_cranberries	= ifelse(cranberries > median_cranberries, 1, 0),

score_avocados	= ifelse(avocados > median_avocados, 1, 0),

score_broccoli	= ifelse(broccoli > median_broccoli, 1, 0),

score_coffee	= ifelse(coffee > median_coffee, 1, 0),

\# 有害成分：< 中位数 → 1 分（严格小于，原始论文 "below the median"）

score_red_meat	= ifelse(red_meat < median_red_meat, 1, 0),

score_processed_meat = ifelse(processed_meat < median_processed_meat, 1, 0),

score_refined_grains = ifelse(refined_grains < median_refined_grains, 1, 0),

\# 高脂饮食：脂肪供能 < 40% → 1 分（固定阈值）

score_high_fat = ifelse(high_fat_diet == 0, 1, 0),

\# 总分（13 项）

DI_GM_score = score_fermented_dairy + score_chickpeas + score_soybeans +

score_whole_grains + score_fiber + score_cranberries +

score_avocados + score_broccoli + score_coffee +

score_red_meat + score_processed_meat + score_refined_grains +

score_high_fat,

\# 亚分

BGMS = score_fermented_dairy + score_chickpeas + score_soybeans +

score_whole_grains + score_fiber + score_cranberries +

score_avocados + score_broccoli + score_coffee,

DGMS = score_red_meat + score_processed_meat + score_refined_grains +

score_high_fat

)

cat("\n=== DI-GM 评分分布（两天平均，5 周期合并）===\n")

cat(sprintf("范围：%d - %d（理论 0-13）\n",

min(di_gm_data$DI_GM_score, na.rm = TRUE),

max(di_gm_data$DI_GM_score, na.rm = TRUE)))

cat(sprintf("均值：%.2f ± %.2f\n",

mean(di_gm_data$DI_GM_score, na.rm = TRUE),

sd(di_gm_data$DI_GM_score, na.rm = TRUE)))

cat(sprintf("中位数：%.1f\n", median(di_gm_data$DI_GM_score, na.rm = TRUE)))

cat("\n数据覆盖情况：\n")

cat(sprintf(" 有两天可靠数据：%d 人（%.1f%%）\n",

sum(di_gm_data$has_day2 == 1, na.rm = TRUE),

mean(di_gm_data$has_day2 == 1, na.rm = TRUE) * 100))

cat(sprintf(" 仅 Day 1（Day 2 用 Day 1 填充）：%d 人（%.1f%%）\n",

sum(di_gm_data$has_day2 == 0, na.rm = TRUE),

mean(di_gm_data$has_day2 == 0, na.rm = TRUE) * 100))

cat("\n各成分得分率：\n")

score_rates <- di_gm_data %>%

summarise(across(starts_with("score_"), ~ mean(.x, na.rm = TRUE))) %>%

round(3)

print(t(score_rates))

\# ----------------------------------------------------------------------------

\## 2.8 输出

\# ----------------------------------------------------------------------------

cat("\n--- 保存结果 ---\n")

di_gm_output <- di_gm_data %>%

select(SEQN, cycle, RIAGENDR, RIDAGEYR,

TKCAL, TTFAT, TFIBE, fat_energy_pct,

WTDR, has_day2,

fermented_dairy, chickpeas, soybeans, whole_grains,

cranberries, avocados, broccoli, coffee,

red_meat, processed_meat, refined_grains,

score_fermented_dairy, score_chickpeas, score_soybeans,

score_whole_grains, score_fiber, score_cranberries,

score_avocados, score_broccoli, score_coffee,

score_red_meat, score_processed_meat, score_refined_grains,

score_high_fat,

DI_GM_score, BGMS, DGMS)

output_xlsx <- "DI_GM_score_5cycles_day1day2.xlsx"

write_xlsx(

list(

DI_GM_Scores	= di_gm_output,

Gender_Medians	= medians,

Score_Rates	= as.data.frame(t(score_rates)),

Cycle_SampleSize = as.data.frame(di_gm_data %>% count(cycle))

),

output_xlsx

)

cat(sprintf("Excel 已保存：%s（%d 名受试者）\n", output_xlsx, nrow(di_gm_output)))

output_csv <- "DI_GM_score_5cycles_day1day2.csv"

write.csv(di_gm_output, output_csv, row.names = FALSE)

cat(sprintf("CSV 已保存：%s\n", output_csv))
