# Supplementary material for: Association between the dietary index for gut microbiota and fracture prevalence among women aged 50 years and older: a NHANES-based study with a preliminary exploratory pilot analysis
Source: Front Public Health. 2026 Oct 6;14:1933908. doi: 10.3389/fpubh.2026.1933908 (PMC13680436; doi:10.3389/fpubh.2026.1933908)
Supplement: Supplementary file 2 [file supplementary_file_2.docx]

**Association between the Dietary Index for Gut Microbiota and Fracture Prevalence among Women Aged 50 Years and Older: A NHANES-Based Study with a Preliminary Exploratory Pilot Analysis**

**Ji Wu^1^, Yunping Liu^2^, Zihao Huang^3^, Xingming Ning^4^, Tao Xu^1^, Qiongxia Yu^1^, Jing Guo^1^, Luming Qi^2^, Zhangmeng Xu^1*^**

^1^ Department of Cervicodynia/Omalgia/Lumbago/Sciatica, Sichuan Province Orthopedic Hospital, Chengdu, Sichuan, China.

^2^  College of Health Preservation and Rehabilitation, Chengdu University of Traditional Chinese Medicine, Chengdu, Sichuan, China.

^3^ College of Pharmacy, Chengdu University of Traditional Chinese Medicine, Chengdu, Sichuan, China.

^4^ Department of Foot and Ankle Surgery, Sichuan Province Orthopedic Hospital, Chengdu, Sichuan, China.

| **Supplementary Table 1. Missing data report** | | | |
| --- | --- | --- | --- |
| **Variable** | **Missing (n)** | **Missing rate (%)** | **Imputed** |
| Total spine BMD | 2,687 | 42.53 | No |
| Femoral neck BMD | 1,280 | 20.26 | No |
| PIR | 611 | 9.67 | Yes |
| Vitamin D | 446 | 7.06 | Yes |
| Alcohol | 335 | 5.3 | Yes |
| BMI | 93 | 1.47 | Yes |
| Education | 11 | 0.17 | Yes |
| Marital status | 6 | 0.09 | Yes |
| Smoking | 3 | 0.05 | Yes |
| Age | 0 | 0 | No |
| Race | 0 | 0 | No |
| Physical activity | 0 | 0 | No |
| Hypertension | 0 | 0 | No |
| Diabetes | 0 | 0 | No |
| Hormone therapy | 388 | 6.14 | Yes |
| Glucocorticoid exposure | 1,086 | 17.19 | Yes |
| DI-GM score | 0 | 0 | No |
| Any fracture | 0 | 0 | No |
| Fragility fracture | 0 | 0 | No |
| Survey design variables (SDMVPSU, SDMVSTRA, WTMEC2YR) | 0 | 0 | No |
| *Note: Missing rate = missing count / total analytical sample (n = 6,318)* × 100. BMD variables were excluded from imputation because of high missing rates and because they were not included as covariates in the primary model. | | | |

| **Supplementary Table 2. Predictor matrix for multiple imputation** | | |
| --- | --- | --- |
| **Variable** | **Imputed** | **Used as predictor** |
| Age | No | Yes |
| Race | No | Yes |
| Marital status | Yes | Yes |
| PIR | Yes | Yes |
| Education | Yes | Yes |
| Smoking | Yes | Yes |
| Alcohol | Yes | Yes |
| Vitamin D | Yes | Yes |
| Physical activity | No | Yes |
| Hypertension | No | Yes |
| Diabetes | No | Yes |
| BMI | Yes | Yes |
| Hormone therapy | Yes | Yes |
| Glucocorticoid exposure | Yes | Yes |
| DI-GM score | No | Yes |
| Any fracture | No | Yes |
| Fragility fracture | No | Yes |
| SDMVPSU | No | Yes |
| SDMVSTRA | No | Yes |
| WTMEC2YR | No | Yes |
| *Note: All variables listed above were included in the imputation model. Every variable was used as a predictor for every imputed variable; a variable did not predict itself (diagonal = 0). Femoral neck BMD and total spine BMD were excluded from the imputation dataset and were not used as predictors.* | | |

Supplementary Figure S1. Trace plots of the multiple imputation chains for variables with missing data.
Note: Each colored line represents one imputation chain (m = 20). The x-axis is the iteration number (1–20); the y-axis is the chain mean or proportion. All chains showed adequate mixing with no visible trends.
